# Incorporating the Heating Stage into Processability Maps for Epoxy Curing Optimization

**DOI:** 10.1021/acsomega.6c02541

**Published:** 2026-07-07

**Authors:** Sihem Zaidi, Daniel Sánchez-Rodriguez, Ahmed Saleh, Jordi Farjas, Paul Spencer, Josep Costa

**Affiliations:** 1 AMADE - Analysis and Advanced Materials for Structural Design, Polytechnic School, 16738 University of Girona, C/ Universitat de Girona 4, Girona E-17003, Spain; 2 GRMT - Materials Research Group and Thermodynamics, Polytechnic School, 16738 University of Girona, C/ Universitat de Girona 4, Girona E-17003, Spain; 3 Materials Engineering Department, Faculty of Engineering, Zagazig University, Zagazig 44519, Egypt; 4 295814 Gurit - Gurit (U.K.) Ltd., St. Cross Business Park, Newport, Isle of Wight PO30 5WU, United Kingdom

## Abstract

Real curing cycles combine heating ramps and isothermal holds, producing trajectories that conventional charts fail to capture. Isothermal time–temperature–transformation charts (TTT) neglect dynamic heating, while continuous-heating-transformation diagrams (CHT) oversimplify by omitting isothermal stages, leading to significant deviations from experimental observations. In this paper, we develop a comprehensive time–temperature–transformation diagram that considers an initial heating ramp and subsequent isothermal stage (TTT-β) to assess the processability of the epoxy resins. This chart features the curing, degradation, vitrification, and gelation kinetics evaluated by isoconversional models, thus they allow determining the optimal time and temperature processing. The reliability of these charts has been validated experimentally. Measurements of the curing degree in a typical epoxy–amine resin indicate that, under heating rates, comparable to those used in industrial processes such as autoclave curing, a non-negligible degree of curing is achieved during the heating ramp, before reaching the isothermal stage. Thus, rendering the information from these diagrams unreliable and highlighting the critical importance of the heating phase. We further examined the effects of thermal degradation and vitrification on the interlaminar shear strength (ILSS), compressive strength (σ_c_), and the compressive modulus (*E*_c_) of the laminate. Our analysis confirms that degradation significantly affects the composite’s mechanical properties and that the proposed charts can support the definition of processing conditions for safe curing.

## Graphical abstract

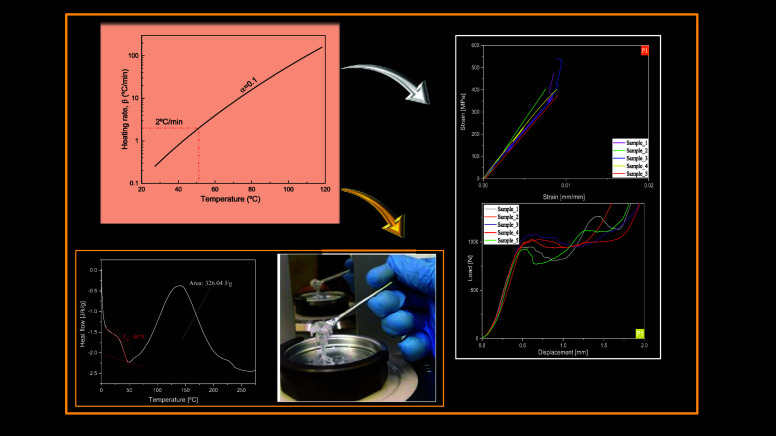

## 1 Introduction

Thermosetting epoxies are key components that hold immense importance across various industries, including construction, automotive, smart coatings, electronic-encapsulating materials^1^ aerospace,^2^ and polymer matrices for fiber-reinforced composites.^3,4^ Their success is attributed to a number of properties such as excellent adhesion, high thermal stability, high resistance to chemicals and solvents, and high thermomechanical performance.^4^ Besides, epoxies offer a favorable balance between performance and cost. Given their good properties and industrial relevance, considerable research efforts have been directed toward optimizing their processing conditions.

Both the cost-effectiveness and performance of epoxy systems are closely linked to the thermal conditions applied during curing.^5,6^ On the one hand, achieving complete cure is essential for maximizing the thermal and mechanical performance of the material. During curing, the thermoset can undergo two major transitions: gelation and vitrification. Both processes derive from the increase in molecular weight as the degree of cure progresses. Gelation marks the point at which cross-linking results in the formation of a continuous network, which irreversibly transforms the material from a viscous liquid into a viscoelastic gel.^7,8^ Vitrification is the transition into the glassy state. It occurs when the glass transition (Tg) approaches the processing temperature, T. As a result, the mobility of the system decreases, and the reaction kinetics is no longer chemically controlled, but diffusion-controlled.^9^ Understanding the kinetics of these two thermally activated transitions is crucial as they impact on the final performance of these materials.

To characterize these transformations, a wide range of experimental and modeling approaches has been proposed for constructing TTT diagrams of epoxy systems. Differential scanning calorimetry (DSC) is widely used due to its ability to monitor reaction heat flow and track cure kinetics under both isothermal and nonisothermal conditions.^10^ When combined with kinetic models such as autocatalytic or Kamal–Sourour, DSC enables the construction of these diagrams for a variety of epoxy chemistries. For instance, diglycidyl ether of bisphenol A (DGEBA) cured with 4,4′-diaminodiphenyl sulfone (DDS), widely used in aerospace composites, typically cures in the range of 120–180 °C and can reach ultimate glass transition temperatures (Tg∞) above 200 °C.^11^ In these systems, vitrification may occur during isothermal holds below approximately 160 °C, significantly slowing the reaction rate due to diffusion limitations.

Similarly, DGEBA systems cured with Dicyandiamide, commonly used in prepregs and electronic encapsulation, have been extensively studied using combined DSC and dielectric analysis (DEA).^12^ These latent systems typically exhibit gelation around 140–160 °C, with final Tg values between 150 and 180 °C depending on formulation and accelerator content. DEA is particularly suited in these cases as it tracks ion mobility and dielectric changes, enabling more precise identification of gelation and vitrification boundaries within TTT frameworks. Epoxy/anhydride systems such as DGEBA cured with methylhexahydrophthalic anhydride have also been widely investigated, where slower curing kinetics in the range of 80–140 °C allow detailed rheological characterization.^13^ In such systems, gelation is commonly determined from the crossover of storage and loss moduli in oscillatory rheometry, while vitrification is associated with the intersection between the evolving Tg and curing temperature.^14^

Beyond these classical systems, epoxy formulations have also been extended to fast-curing resins designed for high-throughput manufacturing processes. These include accelerated DGEBA-based systems and highly reactive amine or anhydride chemistries used in resin transfer molding, vacuum-assisted resin infusion, and injection-based composite manufacturing. In such cases, significant chemical conversion can occur during mold filling or injection, before the material reaches the nominal curing temperature. This leads to premature viscosity build-up and even partial gelation under nonisothermal conditions, thereby violating the assumptions of ideal isothermal curing implicit in conventional TTT analysis. Reported systems can exhibit gelation below 80–100 °C depending on catalyst concentration, while final cure stages may occur at 140–180 °C. Consequently, the curing path becomes highly dependent on thermal history, making the process particularly sensitive to heating rate and residence time during processing.

TTT diagrams for isotherms and CHT for continuous heating diagrams provide an effective means of visualizing the evolution of these thermally activated processes and their transitions, making them powerful tools for identifying optimal processing conditions.^15,16^ Recently, we refined classical TTT diagrams to broaden their applicability by incorporating additional factors such as degradation, overheating, and the occurrence of overshoot and thermal-runaway phenomena in biobased epoxy resins^17^ as well as vitrimer materials.^18,19^

Real curing cycles, however, necessarily involve both heating ramps and isothermal holds, creating an interdependent curing trajectory that neither TTT or CHT diagram can fully capture. The TTT charts, for example, do not account for the progress of the reaction during the ramp that precede isothermal holds, leading to potential inaccuracies in predicting early stage curing behavior. In contrast, CHT diagrams effectively model transformations during continuous heating but neglect the critical isothermal stages. Consequently, the predictions made with these type of diagrams may vary significantly from the experimental results obtained under realistic curing profiles.

To tackle this issue, we have developed a novel diagram that takes into account the heating ramp along with the subsequent isotherm. The key curing transitions namely gelation, vitrification and thermal degradation were examined through thermal analysis. Our study focuses on Gurit’s Prime130, a commercial epoxy resin based on DGEBA, which accounts for over 90% of today’s thermoset epoxy production.^20^ Its widespread use makes it a highly representative material for industrial applications. Additionally, the mechanical properties of fiber reinforced epoxy laminate resulting from different thermal histories were investigated, allowing the correlation of the results with the thermal process predicted by the diagram. The findings confirm the reliability of the proposed diagrams.

## 2 Methodology

### 2.1 Materials

We used Gurit’s commercial epoxy Prime130, a formulation based on DGEBA epoxy groups, cured with the hardener isophorone diamine (IPDA), both supplied by Gurit. The two components were neatly mixed according to the manufacturer’s instructions, at a ratio of 27 phr (parts of hardener per parts of resin), and no additional purification steps were taken.

For reinforcement, unidirectional (UD) glass fiber (UD 8092) with an areal density of 625 g/m^2^ was supplied by MEL COMPOSITES, S.L. (Barcelona, Spain). The fabric is composed of approximately 99% glass fiber and incorporates a small amount of thermoplastic polyester stitching yarns (≈1%), primarily located in the selvage region. These yarns enhance edge stability, improve handling, and prevent fraying during processing. Supplied as a raw tape, the material exhibits a nominal weight of 625 g/m^2^ (±5%) and a thickness of about 0.42 mm in the central region and 0.44 mm at the edges, indicating a uniform and compact weave. In accordance with CEI EN 61067–2, the fiber architecture comprises a warp density of 2.5 ± 0.2 threads/cm and a weft density of 1.5 double threads ± 0.2 threads/cm, where the weft direction incorporates thermoplastic yarns to improve thermal and dimensional stability.

### 2.2 Thermal Analysis

To analyze the curing reaction behavior, we conducted measurements using a TA Instruments Q2000 DSC. The tests were performed under dynamic heating conditions, applying rates between 1.25 and 20 °C/min across a temperature interval of 0–290 °C. To minimize premature curing prior to DSC measurements and ensure reproducible initial conditions, the sample was prepared following a consistent protocol. After mixing the hardener and the epoxy, the resin system was immediately transferred into DSC crucibles (enough number to perform the required heating rates). The elapsed time between mixing and the start of the DSC experiment was kept as short as possible (typically less than 1 min). According to the kinetic model, achieving a degree of cure of 0.001 at 25 °C requires approximately 70 s, a value that decreases to 45 s at 30 °C. Thus, the degree of cure accumulated prior to measurement is negligible. Uncured samples were saved on the freezer up to −32 °C to avoid any premature curing. DSC experiments were performed in a nitrogen atmosphere maintained at 50 mL/min. The total reaction heat was quantified by integrating the exothermic signal of the DSC curve using a straight-line baseline.

To investigate the relationship between the Tg and the degree of cure, α, a series of samples were cured at a heating rate of 5 °C/min to reach various degrees of cure according to the nonisothermal predictions performed with the Friedman isoconversional kinetic analysis (Section 2.5). Tg, was determined following the methodology described by E.A. Turi.^21^ Using this approach, the Tg of the fully cured epoxy resin, T_g∞_, was determined to be 135.7 °C, in good agreement with the T_g∞_ = 133 °C reported by the manufacturer. Using the ASTM E-1269 procedure,^22^ a heat-capacity value of 1.4 J/g/K was obtained for the fully cured sample.

A Mettler Toledo TGA/DSC1 thermobalance was utilized in thermogravimetric analysis (TGA) to gauge the resin’s thermal stability. Over a temperature range of 50 to 800 °C, completely cured specimens have been heated at rates between 1.25 °C/min and 20 °C/min. Each specimen, approximately 4–5 mg was put into 150 μL uncovered Al_2_O_3_ crucibles. A constant flow of synthetic air at 50 mL·min^–1^ was set during the TGA measurements. The liquid resin was placed on the hot plate at room temperature and then heated up to 120 °C, held for 10 min, and subsequently postcured at 140 °C for 1 h. After curing, the samples were directly subjected to TGA measurements starting from 50 °C. The cured specimens were stored in a hermetically sealed bag to prevent moisture absorption prior to testing. Furthermore, all TGA curves remained stable up to approximately 250 °C before the onset of degradation, indicating negligible moisture content in the samples.

To monitor the resin’s viscoelastic characteristics evolved across the various stages of curing, we used Anton Paar rheometer MCR 302e model. The uncured samples were treated at temperatures ranging from 80 to 100 °C under rotational oscillation shear using 25 mm diameter aluminum disposable parallel plate. To minimize premature curing, the target temperature was reached at the maximum available heating rate, bringing the system from 20 °C to the set point in less than 1 min. Any residual curing occurring during this heating phase results in a minor deviation from ideal isothermal conditions. The experiments maintained a constant frequency of 1 Hz and a consistent shear strain of 0.5%. The samples were subjected to the testing with a fixed gap of 1 mm. In alignment with the methodology outlined in,^23,24^ our approach aimed to determine the gelation evolution by identifying the crossover point between the storage modulus (G’) and the loss storage modulus (G’’) derived from the isothermal scans.

### 2.3 Composite Manufacturing

Four plies of the glass fiber were stacked together and 4 thermocouples were used to monitor the temperature evolution (Figure 1b). Thermocouple T1 was placed at the midplane of the laminate. Thermocouple T2 and T4 were located on one surface of the glass plate. Thermocouple T3 was installed inside the oven to monitor and control its temperature. The glass-reinforced epoxy laminates were produced by vacuum infusion: a vacuum pump is installed to eliminate air bubbles and ensure that the resin is properly distributed in the fabric, once the air has been extracted from the material, the epoxy resin, premixed with its hardener, is introduced into the system through an inlet pipe under vacuum pressure. When the resin is fully permeated through the composite reinforcement, the excess resin passes through the outlet pipe. Four composite laminates (P1, P2, P3, and P4) were cured in an oven using different thermal cycles, starting from 25 °C and following the temperature programs shown in Table 1. The thermal cycles were intentionally designed to produce laminates with distinct resin states (fully cured, vitrified, and degraded) in order to investigate their influence on the mechanical properties of the manufactured panels. A constant heating ramp of 2 °C/min was adopted for all curing conditions to ensure controlled and homogeneous temperature evolution throughout the laminate thickness while minimizing thermal gradients and residual stresses. The P1 cycle (140 °C for 20 min) corresponds to the manufacturer-recommended curing window and was selected to achieve a near complete degree of cure (α ≈ 1) without inducing thermal degradation. In contrast, P2 (155 °C with no isothermal dwell) was designed to promote full curing mainly during the heating stage due to the higher peak temperature, allowing assessment of whether rapid curing produces mechanical properties comparable to the reference fully cured condition. The P3 cycle (100 °C for 60 min) was intentionally selected to induce vitrification; at this relatively low temperature, the Tg approaches the curing temperature before complete cross-linking occurs, thereby restricting molecular mobility and slowing the curing reaction, resulting in a partially cured vitrified state (α ≈ 0.86). Finally, P4 (170 °C for 390 min) was chosen to induce resin degradation through prolonged exposure to elevated temperature. The combination of high temperature and extended dwell time promotes thermooxidative and thermal degradation mechanisms, leading to significant deterioration of the polymer network and a very low effective conversion state. Dimensions of the glass were chosen as follows: 170 × 140 mm.

**Figure 1:**
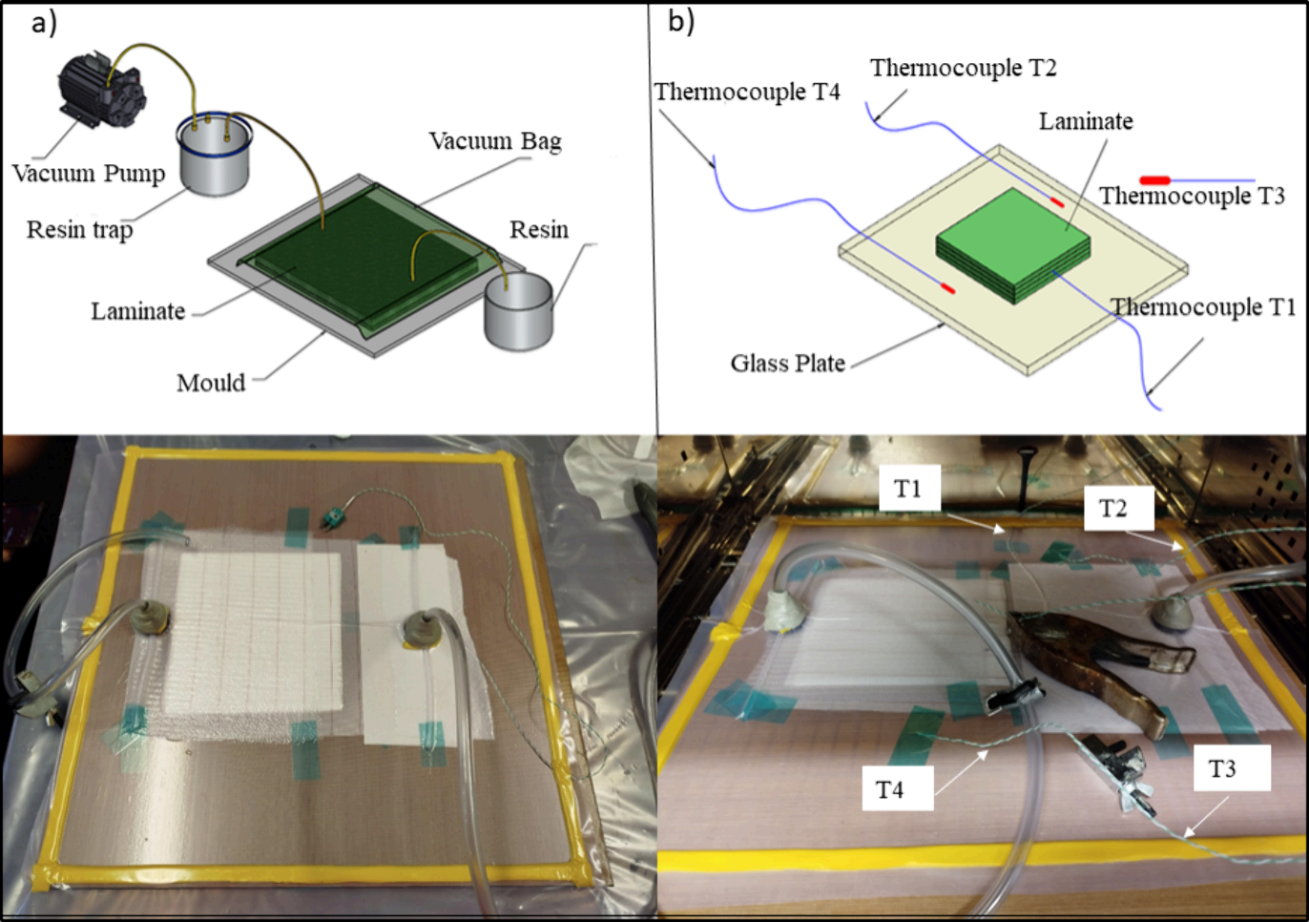
(a) Experimental schematic diagram of the infusion process. (b) Thermocouple placement for temperature monitoring: T1 (laminate), T2 (glass plate), T3 (oven control), and T4 (glass plate).

**Table 1:** Curing Cycles for Laminate Fabrication: P1, P2, P3, and P4

sample	heating ramp [°C/min]	isotherm T [°C]	isotherm time [min]	expected resulting state	expected conversion degree, α
P1	2	140	20	fully cured	1
P2	2	155	0	fully cured	1
P3	2	100	60	vitrified	0.86
P4	2	170	390	degraded	0.015

### 2.4 Mechanical Testing

Interlaminar shear strength (ILSS) tests (short beam) were conducted according to EN2563–1997 standard specification, using unidirectional glass fiber-reinforced composites.^25^ Specimens with 10 mm in width, 20 mm in length, and 2 mm in thickness were tested at room temperature using an MTS insight 50 kN testing machine. In the setup shown in the Supporting Information Figure S1, the specimen rests on two cylindrical supports while a cylindrical head applies a central transverse load. The force is incrementally increased until initial failure is detected. The load at failure is then used to determine the apparent interlaminar shear strength of the composite. Here constant crosshead displacement rate of 1 mm/min was used. The maximum apparent shear strength, τ, in the midplane of the composite was approximated by the Euler–Bernoulli beam theory:

$$\tau_{\max}=\frac{3P_{\max}}{4bh}$$ 1

Where *P*_max_ is the force at failure, *b* is the width of the specimen, and *h* is the thickness of the specimen.

Compression tests were carried out at room temperature on a MTS 25 kN Bionix2 hydraulic testing machine with a load range of 25 kN, following ASTMD6641 method.^26^ Specimens of 140 mm × 13 mm × 2 mm were evaluated (Figure S3). Strain gages of 3 mm were applied at the center of the gauge section on one side for three samples and both sides for two specimens per panel (Figure S2). The tests were performed at a loading rate of 1.3 mm/min. When the specimens were fractured, the test was considered completed.

The compressive strength was calculated by dividing the ultimate load by the specimen cross section according to the following formula:

$$\sigma_{c}=\frac{P}{A}$$ 2

Where σ_c_ is the compressive strength, *P* the load and *A* is the cross-sectional area of the specimen.

### 2.5 Isoconversional Kinetic Analysis

This study exploits the same broad framework described in refs 17 and 18 in order to predict the kinetic parameters, including the pre-exponontial term *Af*(α) and the apparent activation energy, *E*_α_. By applying Friedman’s isoconversional methodology,^27,28^ the kinetic evolution was calculated by utilizing Farjas and Roura’s method^24^ to integrate the transformation rate, dα/d*t*, over time.^29^

$$\alpha_{j+1}=\alpha_{j}+A_{\alpha }f(\alpha_{j})\exp\left(-\frac{E_{\alpha j}}{RT_{j}}\right)\Delta t$$ 3

Here, for a given degree of transformation α_*j*_, *A*_α_ *f*(α_*j*_) and *E*_α*j*_ denote the corresponding discrete numerical values. Equation 3 is valid for any thermal history, allowing its application to isothermal, constant heating rate, or combined heating-isothermal curing programs.^30^

### 2.6 Construction of the Processing Charts

#### 2.6.1 Curing Kinetics

Figure 2 shows how the heat-flow signal varies with temperature for the different heating programs applied, which ranged from 1.25 to 20 °C/min. All curves exhibit a characteristic exothermic event associated with the cure reaction of the material and indicate that the epoxy–amine system exhibits an exothermal signal already at ambient temperatures, suggesting that the curing reaction may initiate slowly at ambient conditions. This behavior is consistent with the intrinsic reactivity of epoxy–amine systems. Furthermore, the total heat generated during this process was calculated by integrating the area under this peak after subtraction of a baseline. Across all heating rates, the average reaction enthalpy was 438 ± 7 J/g, while the detailed values for each experiment are compiled in Table S1. Deviations in the enthalpy values obtained from different experiments remain below 2%, confirming that the baseline selection procedure is consistent and reproducible across the full set of measurements.

**Figure 2:**
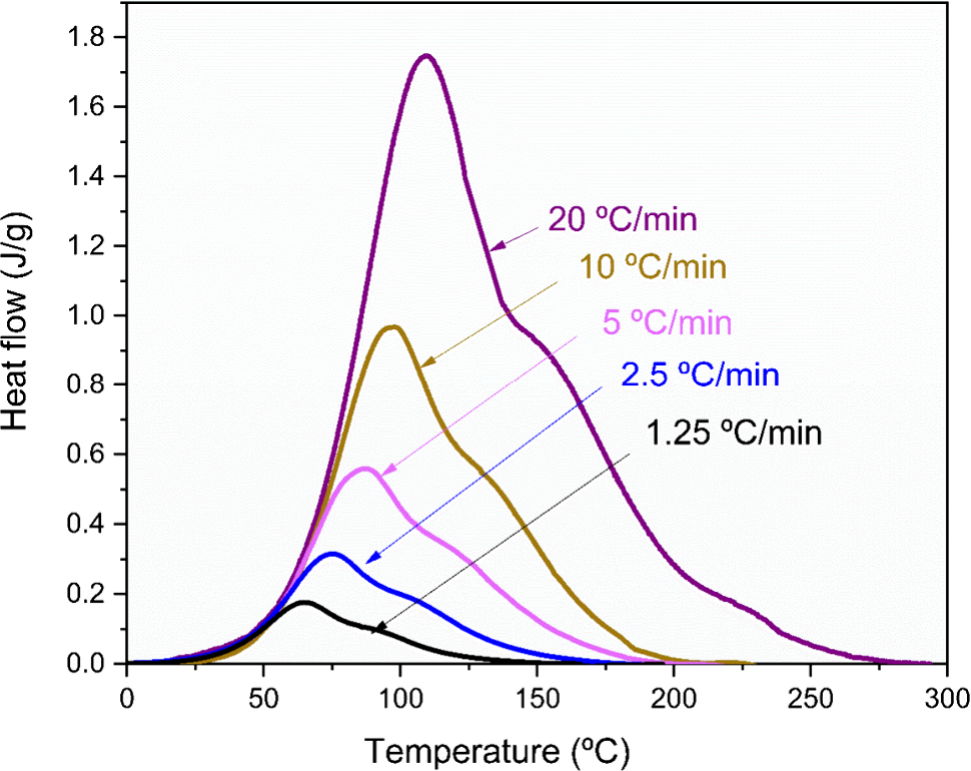
DSC analyses conducted at various heating rates revealed the resin’s nonisothermal curing behavior.

Figure 3 illustrates the evolution of the curing conversion with time. The degree of conversion was calculated from the integrated area of the DSC exothermic peak shown in Figure 2 relative to the total reaction enthalpy after baseline subtraction, according to:.

$$\alpha (T)=\frac{\Delta H_{T}}{\Delta H_{\mathrm{total}}}$$ 4

**Figure 3:**
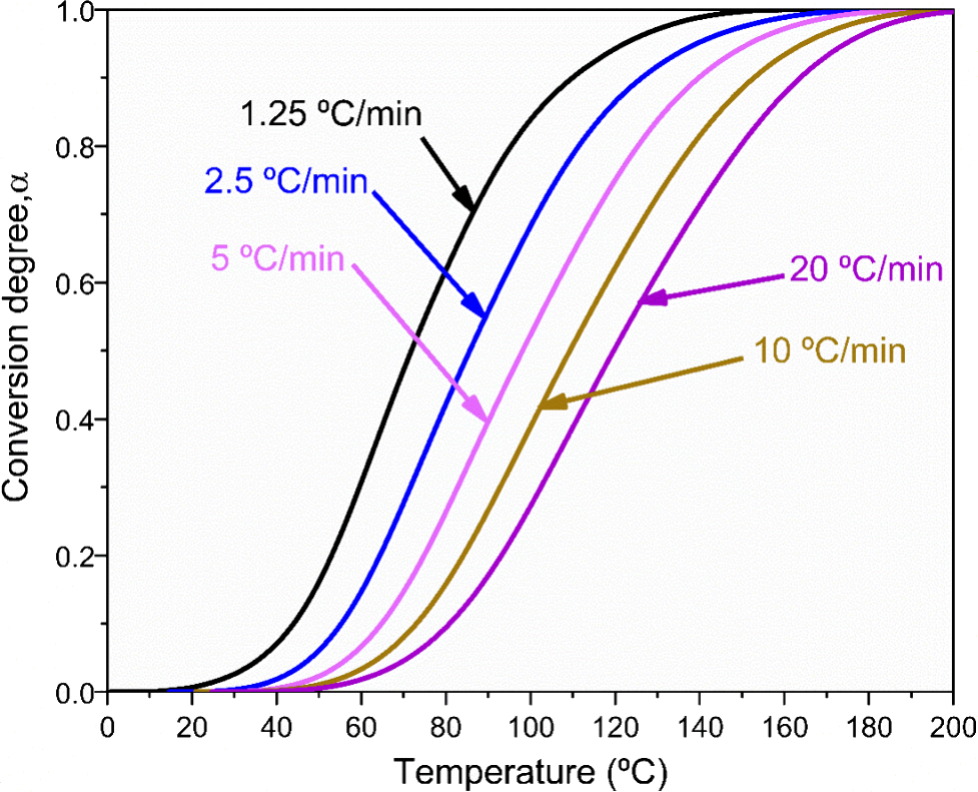
Degree of cure as a function of temperature derived from the integration of DSC curves.

To obtain a deeper understanding of the underlying kinetic behavior, the Friedman differential isoconversional method was applied to the DSC data sets presented in Figure 2. The analysis was performed using the logarithmic form of the Friedman equation:

$$\ln\left(\frac{d\alpha }{dt}\right)=\ln[Af(\alpha )]-\frac{E_{\alpha }}{RT}$$ 5

This approach provides the apparent activation energy, *E*_α_, at different degrees of curing without imposing a predefined kinetic model. Figure 4 plots the calculated *E*_α_ values against the extent of cure, revealing a very strong correlation, with a coefficient of determination close to unity. This high R^2^ value indicates that the model-free analysis is consistent and accurately reflects the reaction progression throughout the conversion range investigated.

**Figure 4:**
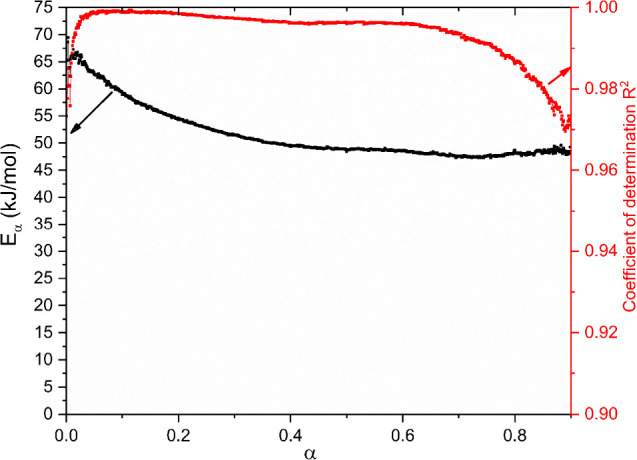
Apparent activation energy, *E*_α_, as a function of on the degree of cure, α.

The curing reaction of a DGEBA with a diamine involves two main steps: Initially, a primary amine reacts with an epoxy group to form a secondary amine and hydroxyl groups. In the second step, the newly formed secondary amines react with additional epoxy groups, yielding tertiary amines, thereby increasing the cross-link density within the polymer network.^25,26^ The large values of *E*_α_ at low conversion degree (α < 0.1) can be attributed to the primary amine addition (activation energies between 58 and 66 kJ/mol have been reported for the primary amine addition.^31,32^ For α > 0.1 the apparent activation energy drops due to the catalytic effect of the hydroxyl groups, stabilizing at approximately 50 kJ·mol^–1^. This value is consistent with the activation energies typically reported for DGEBA–diamine curing reactions.^33,34^

As Tg approaches the processing temperature during isothermal curing, the reaction becomes diffusion-limited. Well known as vitrification in which polymer chains become less mobile and may even totally freeze. Therefore, monitoring Tg is crucial to ensure optimal curing. This dependence is well described by the DiBenedetto relationship.^35,36^

$$\frac{T_{g}-T_{g_{0}}}{T_{g_{\infty }}-T_{g_{0}}}-\frac{\lambda \alpha }{1-(1-\lambda )\alpha }$$ 6

Where α is the conversion degree, *T*_g0_ refers to Tg of the uncured sample, *T*_g∞_ denotes Tg of the completely cross-linked network and λ is a parameter controlling the convexity of the dependence that ranges between 0 and 1. Figure 5 illustrates the Di-Benedetto curve obtained after adjusting the DSC data to the eq 4, note that the fitted value of λ = 0.57 falls within the typical range (0.5–0.6) observed for many epoxy resin systems.^37^

**Figure 5:**
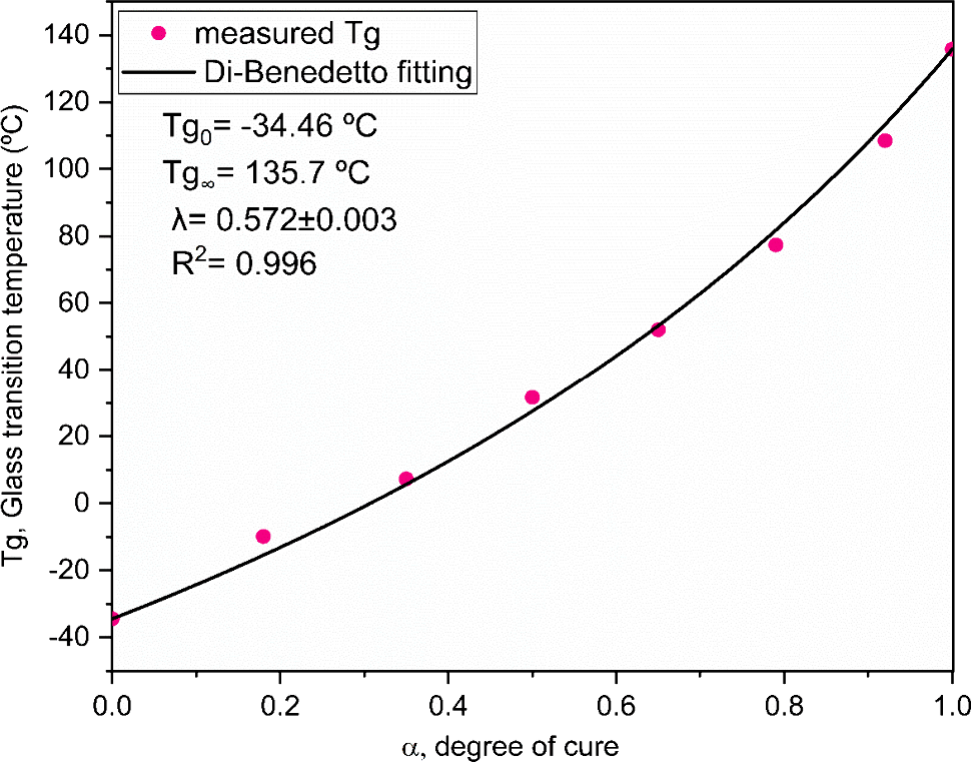
DiBenedetto curve fitting for the Prime 130 epoxy resin.

In the present work, to identify the gel time, isothermal rheological time sweep experiments were performed. These experiments consist on tracking the evolution of the storage modulus (*G*’) and the loss modulus (*G*’’) over time using low-amplitude oscillatory shear at a constant frequency. As shown in Figure 6, during the early stages of the curing, the sample is in a liquid state characterized by *G*’’ > *G*’. The two moduli rise with the cross-linking density and molecular weight of the epoxy-hardener system. At the gel point, the system forms continuous network, leading to the crossover of the storage modulus (*G*’) and the loss modulus (*G*’’).^38^

**Figure 6:**
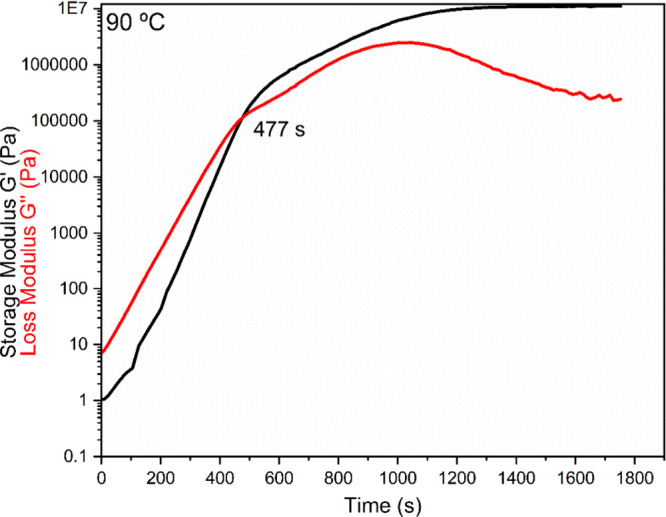
Isothermal rheological time sweep experiment of the epoxy resin Prime 130.

The gel times t_gel_ and the conversion degree,α_gel_ can be determined by Freidman kinetic analysis. The degree of cure at the gel point is α_gel_ = 0.59 which agrees with reported values for similar systems, between 50 and 60%.^39^

The three temperatures and gel times can be used to determine the gelation activation energy, *E*_gel_,^40^

$$t_{\mathrm{gel}}=A\exp^{\left(E_{\alpha }/RT\right)}$$ 7

Which can be linearized to

$$\ln(t_{\mathrm{gel}})=\ln(A)+\frac{E_{\mathrm{gel}}}{R}+\left(\frac{1}{T}\right)$$ 8

By constructing an Arrhenius plot using the measured gel times, t_gel_ and the corresponding inverse temperatures, a linear relationship emerges, from which an activation energy *E*_gel_ of 57.7 kJ/mol is determined (see Figure 7).

**Figure 7:**
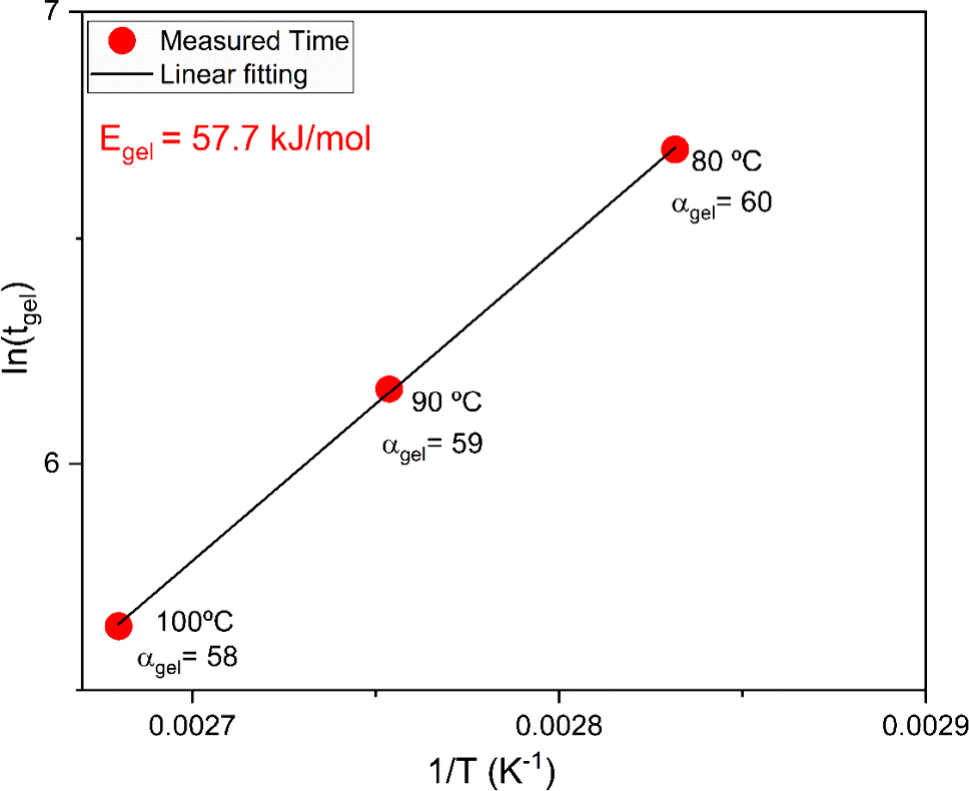
Arrhenius plot of gel time for curing kinetics.

To provide a theoretical reference for the experimentally determined gelation behavior, the Flory–Stockmayer statistical theory of step-growth polymerization can be considered. In this framework, gelation occurs when the formation of an infinite network becomes statistically possible based on the functionality of the reacting species.

For an ideal stoichiometric system of two monomers, *A*_*f*_ and *B*_*g*_, the critical extent of reaction at gelation is given by:

$$\alpha_{\mathrm{gel}}=\frac{1}{[(f-1)(g-1){]}^{0.5}}$$ 9

where *f* and *g* represent the functionalities of the epoxy and hardener, respectively. For the DGEBA–IPDA system, considering *f* = 2 and *g* = 4, the theoretical gel conversion is approximately α_*gel*_ ≈0.577. This value is in very good agreement with the experimentally determined gel conversion, indicating that gelation in the present system is primarily governed by network connectivity as described by classical step-growth polymerization theory. Minor deviations between theoretical and experimental values may arise from nonidealities such as unequal amine reactivity, cyclization reactions, and diffusion limitations during curing.

#### 2.6.2 Thermal Stability

The degradation kinetics of the fully cured epoxy were investigated via nonisothermal TGA in combination with the thermal mass loss criterion. Figure 8 presents the TGA curves adjusted for various heating rates in air atmosphere. All the TGA curves exhibit at least three degradation steps. However, to develop a processing map, the focus should be put on the first stage of degradation, as it determines the maximum processing temperature. To establish a common physical state across all experiments corresponding to 100% process conversion, total deterioration was defined as the stage at which the material’s mass is effectively reduced to zero.^18,41^

**Figure 8:**
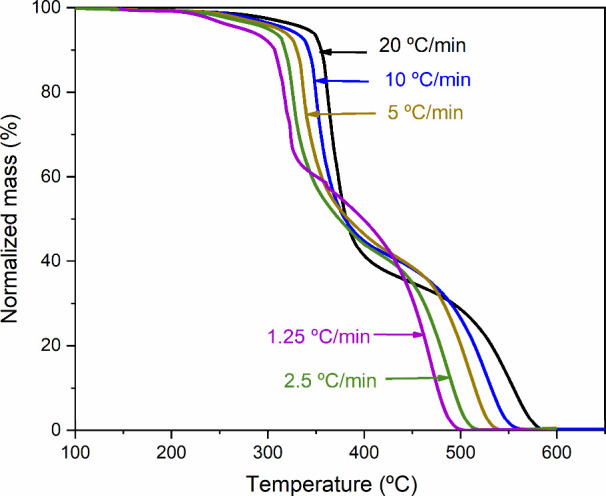
Variation of the normalized mass with temperature under different heating rates.

The kinetic parameters of the early degradation stages were determined by applying Friedman’s method to the TGA curves presented in Figure 8. As depicted in Figure 9, within the 0 < α < 0.05 range,*E*_α_ rises from an initial value of 84 kJ/ to around 210 kJ/mol. The high coefficient of determination (R^2^ above 0.97) for any degree of conversion confirms the reliability of the kinetic analysis.

**Figure 9:**
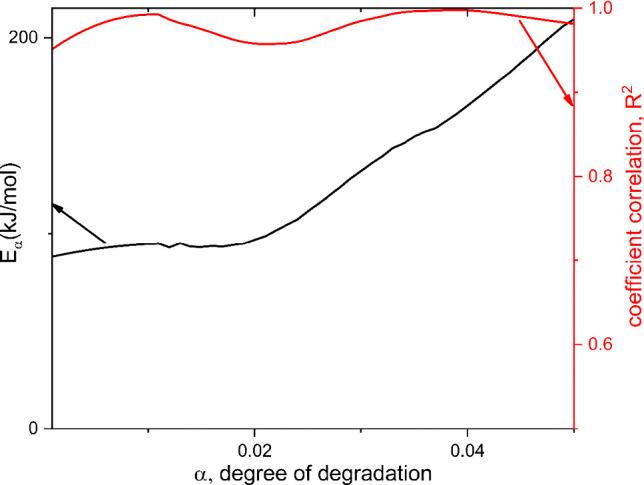
Friedman isoconversional evaluation of the apparent activation energy as a function of α.

#### 2.6.3 Isothermal Processing Chart: TTT

In continuation of our previous work,^12,13^ we have proposed a TTT chart to determine the optimal isothermal processing conditions for the studied commercial epoxy resin. Such a chart is based on the curing and decomposition kinetic analyses detailed in sections[Sec sec2.6.1] and 2.6.2, respectively, and incorporates vitrification, gelation, and thermal degradation. It illustrates the evolution of these phenomena as a function of time at a constant temperature covering a temperature range from 0 to 250 °C. Figure 10 shows in black, the time needed to accomplish a 10, 20, 50, 90, 95, 99, and 100% degree of cure.

**Figure 10:**
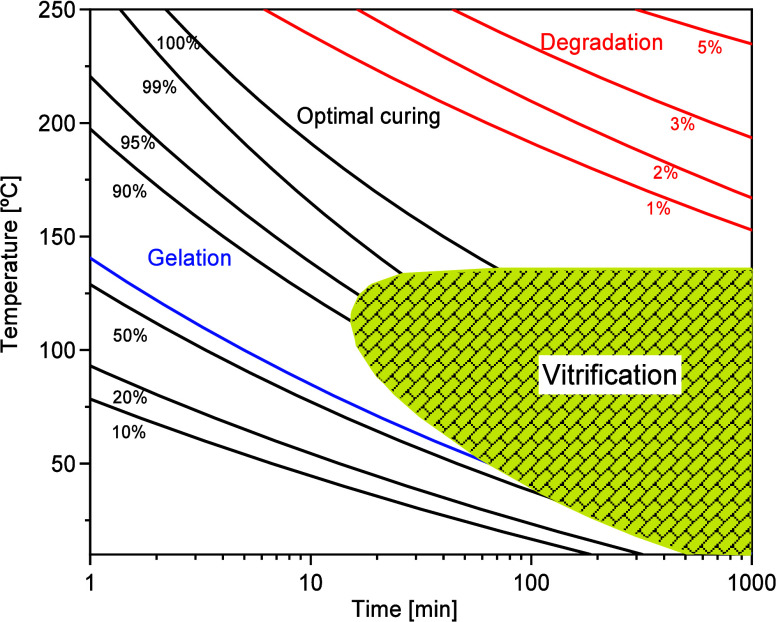
Processing map of the studied epoxy for isothermal conditions: solid black lines illustrate the time needed to complete 10, 20, 50, 90, 95, 99, and 100% of curing. Red solid lines indicate degradation degrees 1, 2, 3, and 5%. The vitreous state of the epoxy resin appears as the yellow dashed area. The solid blue line indicates gelation.

Likewise, the time to vitrification under isothermal conditions was estimated from the evolution of Tg as a function of the degree of cure. The vitrification line defines the boundary beyond which the material transitions into the glassy state (highlighted in yellow dashed region). Notably, at temperatures below 50 °C, vitrification occurs within more than one hour and before the gel point is reached. Isothermal curing below this temperature is therefore not recommended, as the desired mechanical properties cannot be achieved without a subsequent postcure above the vitrification temperature. Further, the degree of conversion for gelation, α_gel_, is highlighted in blue solid line. At 113 °C the prediction indicates that the gelation point is exceeded after just 2.9 min of treatment, and vitrification is achieved within 16 min. At the upper end of the temperature range, thermal degradation may become a concern. The red solid curves indicate isothermal conditions corresponding to relative mass losses of 1 to 5%, providing an upper bound for acceptable processing temperatures and times. When all this phenomenology is combined in a single TTT diagram, researchers can define the isothermal curing conditions (time, t, and temperature, T) that provide the targeted degree of cure without exceeding acceptable degradation thresholds.

#### 2.6.4 Processing Chart Accounting for Heating and Isotherm: TTT-β

Isothermal diagrams are an idealization because they assume that the treatment temperature is reached instantaneously. This is a good approximation if the heating rate is sufficiently high to attain the constant temperature with a negligible degree of cure is accumulated before reaching the target temperature. As discussed in Section 2.6.3, the TTT diagram, Figure 10, shows that isothermal curing below 50 °C is not practical, since the resin vitrifies within approximately one hour at that temperature, effectively arresting the curing process.

Within this context, Figure 11 addresses the following question: for which heating rates does the cure accumulated during the heating ramp become nonnegligible? The *x*-axis shows the target isothermal temperature, and the *y*-axis shows the heating rate at which a degree of cure of 0.1 is reached upon heating to that temperature. The 10% value is adopted as a benchmark for identifying conditions under which ramp effects are significant.

**Figure 11:**
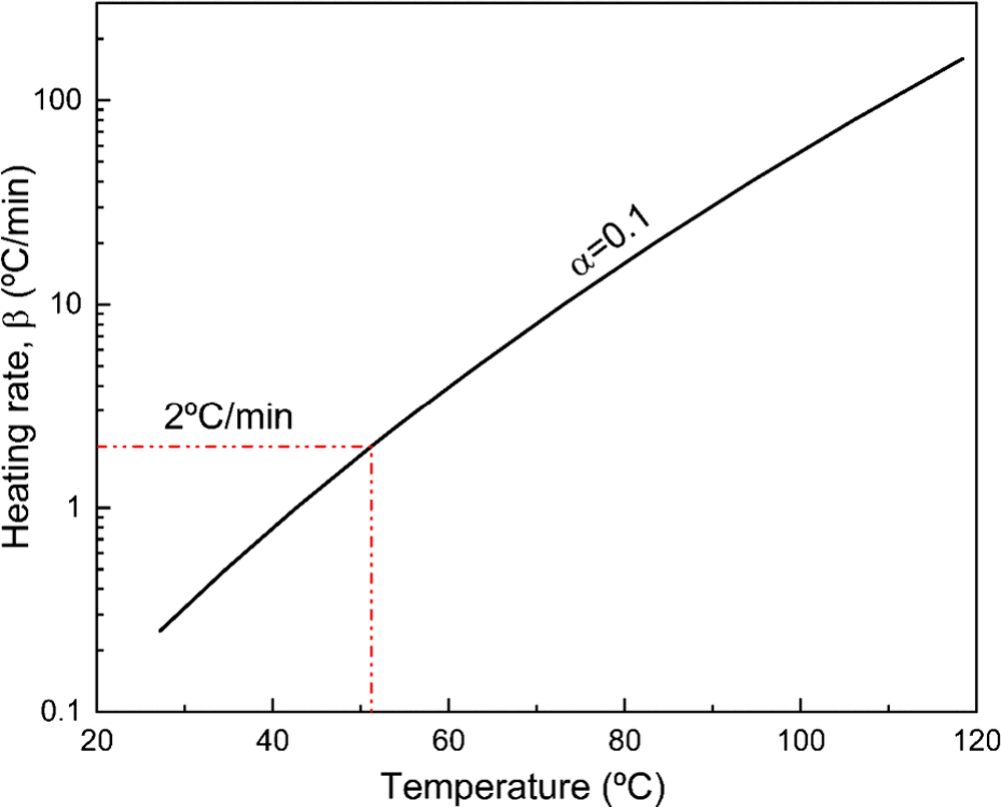
Heating rate at which a degree of cure of 0.1 is reached as a function of the target isothermal temperature.

Figure 11 shows that for a heating rate of 2 °C/min, a representative value for industrial equipment such as autoclaves, which are limited by their high thermal inertia, the 10% threshold is crossed at a temperature only slightly above 50 °C. This means that across the entire practical curing temperature range, the degree of cure accumulated during the ramp exceeds 10% at this rate and grows with increasing target temperature. Lower heating rates would be even more problematic. Consequently, we have developed a TTT-β diagram that accounts for the initial heating stage.

In Figure 12, we show the TTT-β diagram for a heating rate of 2 °C/min. Specifically, this diagram represents the time required for the resin to reach a given degree of cure during the isothermal stage, when that temperature has been reached through constant heating at 2 °C/min. Note that the time shown in Figure 12 does not include the time spent during the constant-rate heating phase; time begins counting from the moment the target temperature of the isothermal treatment is reached.

**Figure 12:**
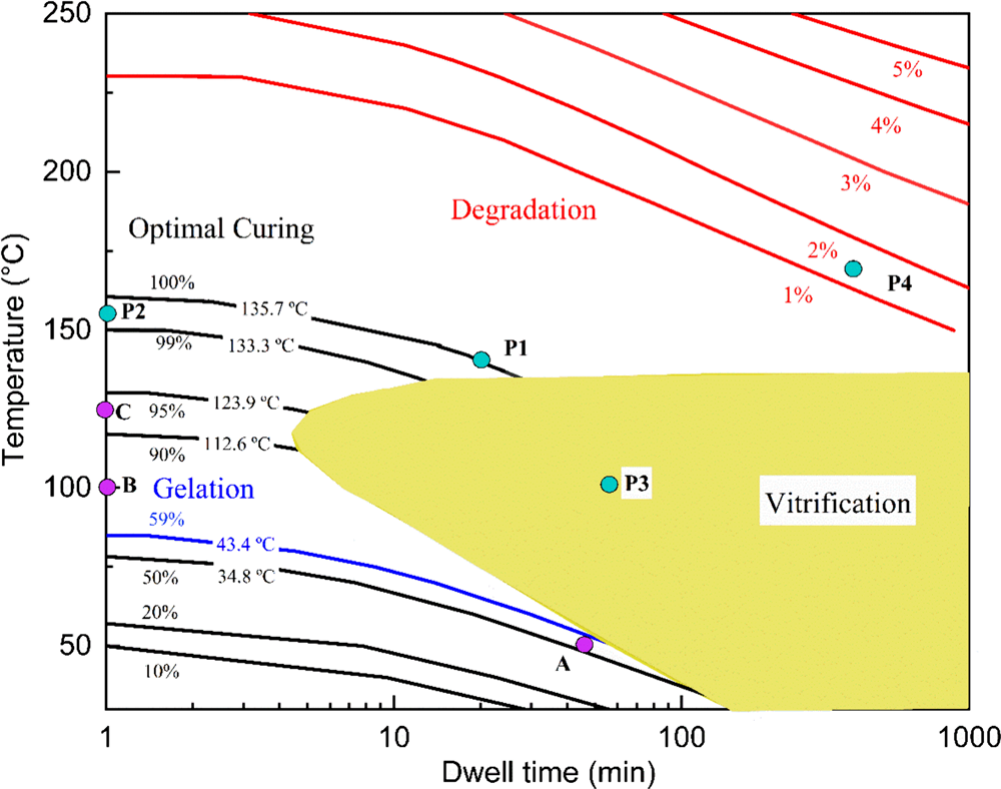
Processing chart taking into account a 2 °C/min heating ramp followed by an isothermal phase: solid black lines indicate the dwell time required to reach 10, 20, 50, 90, 95, 99, and 100% cure. The continuous red lines represent degradation of 1, 2, 3, 4, and 5%. The vitreous state appears as a green dotted line. The solid blue line indicates gelation.

The construction of these diagrams relies on the Friedman’s isoconversional method. As expected, this chart differs from the classical isothermal scenario. For example, a degree of cure of 99% can be achieved at 150 °C during the heating ramp phase, before the isothermal stage begins. Whereas the classical isothermal TTT diagram would predict that the sample must be held at that temperature for more than 10 min to reach the same conversion. This clearly illustrates how neglecting the heating stage can lead to substantial inaccuracies.

The TTT-β diagram highlights two key trade-offs in the curing process. At intermediate temperatures, the combination of a controlled heating ramp and a subsequent isothermal hold produces a kinetic synergy: the ramp initiates the reaction, while the isothermal stage allows the system to progress toward higher degrees of cure without overshooting. At higher temperatures (above 220 °C), however, the situation becomes more constrained. Although elevated temperatures can drive the reaction to completion in a shorter time, they also increase the risk of thermal degradation, as shown by the rising degradation thresholds in the diagram. Consequently, both the dwelling temperature and the cumulative dwell time must be carefully optimized. Excessive temperatures or prolonged dwell times can compromise material properties through chain scission or oxidative damage, negating the benefits of rapid curing. Thus, the curing strategy must balance fast curing against the need to preserve long-term stability and performance.

This analysis defines the upper limit of the processing temperature range, with the lower limit set by the vitrification phenomenon. In the glassy state, molecular mobility is highly restricted, so under isothermal conditions, the curing rate is typically very slow or essentially negligible.^42^ Purely isothermal treatments thus present two contrasting challenges depending on the chosen temperature. At high temperatures, the large initial gap between the treatment temperature and the instantaneous Tg drives a rapid and potentially difficult-to-control reaction. At low temperatures, Tg rises as curing proceeds until it equals the treatment temperature, at which point vitrification arrests the reaction and prevents full cure from being achieved.

A heating ramp followed by an isothermal dwell can mitigate both of these issues. During the ramp, curing takes place at relatively low temperatures where the reaction rate is moderate and manageable, while the continuous increase in temperature keeps T above the instantaneous Tg throughout the heating stage, thereby preventing vitrification. These considerations motivate the development of a TTT-β diagram that explicitly accounts for the initial heating stage.

## 3 Experimental Validation

### 3.1 Degree of Cure

To experimentally validate the TTT-β diagram, three samples of epoxy resin (A, B, and C) each with a mass of approximately 7 mg (details in Table 2). They have been treated with different thermal histories based on the TTT-β in order to achieve different degrees of cure α. Afterward, DSC analysis was performed to determine their Tg. In fact, Figure S4 in the Supporting Information shows the DSC curves recorded during processing and posterior determination of Tg. The predicted degree of cure for sample A is 0.52 which is below the gelation (α_gel_ = 0.59) and a Tg of 31 °C. The measured Tg was 40 °C associated with a degree of cure of 0.54. Hence, the resin is still in the liquid phase and shows a honey-like rheological behavior (Figure 13). The observed rheological properties are in good agreement with the predictions. In addition, samples B and C were processed at higher temperatures, with the aim of achieving a degree of cure superior to gelation (α ≥ α_gel_). In the case of sample B, the predicted α would be 0.74 and the associated Tg should be at 72 °C. Specifically, sample B attained an α of 0.73, which corresponds to Tg of 77.2 °C. Furthermore, the sample C should have a degree of crosslinking of 0.92 and a Tg of 113 °C. Experiments gave an α of 0.92 and a Tg of 113.5 °C. Overall, all these results match well the predictions.

**Table 2:** Predicted and Measured Properties for Different Temperature Programs

	thermal history			predicted values		measured values	
sample	heating ramp [°C/min]	isotherm [°C]	dwell time [min]	Tg [°C]	α	Tg [°C]	α
A	2	50	45	Tg,_gel_ ∼ 31	0.52	40	0.54
B	2	100	0	Tg ∼ 72	0.74	77.2	0.73
C	2	125	0	Tg ∼ 113	0.92	113.5	0.92

**Figure 13:**
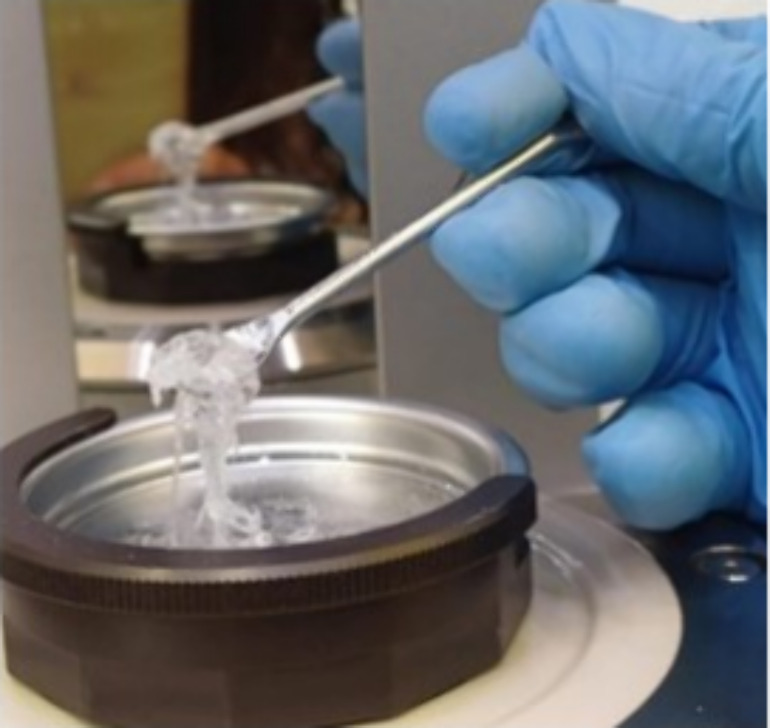
State of the sample A treated following the thermal history indicated in Table 2.

### 3.2 Mechanical Properties

In order to validate that the mechanical properties of a composite laminate correlate well with the degree of cure and degradation of the resin predicted with the TTT-β processing charts, we have measured four key properties: the interlaminar shear strength (ILSS), the compressive strength, the compressive modulus, and Tg under different cure cycles (see details in Table 3). These properties are highly sensitive to the matrix state. For instance, both laminates P1 and P2 are expected to achieve a degree of cure of 100%. P3 was treated aiming to reach the vitrification region. Finally, panel P4 was treated in the region where a mass loss due to thermal degradation between 1 and 2% is expected. Typical load–displacement curves of these samples are provided in the Supporting Information in Figure S5 while compressive stress vs strain curves of epoxy/glass fiber composites treated at different curing cycles are shown in Figure S7.

**Table 3:** Comparative Analysis of Tg, Interlaminar Shear Strength (ILSS), and Compressive Properties of Laminates P1–P4

sample	heating ramp [°C/min]	isotherm [°C]	dwell time [min]	measured Tg [°C]	ILSS average [MPa]	compressive strength [MPa]	compressive modulus [GPa]
P1	2	140	20	130.4	39.1 ± 2.0	442.8 ± 66.8	45.0 ± 2.0
P2	2	155	0	133	42.9 ± 1.1	443.8 ± 38.6	41.9 ± 2.1
P3	2	100	60	116.5	34.7 ± 1.3	354.8 ± 11.5	40.6 ± 3.9
P4	2	170	390	134	17.6 ± 1.4	261.2 ± 44.3	38.8 ± 2.6

Furthermore, Tables S2 and S3 in the Supporting Information summarize the dimensions of each specimen along with their ILSS and compressive properties. Whereas the coefficient of variation was below 8% for all sample conditions.

Figure 14 presents the absolute Tg values in Kelvin, along with absolute values of ILSS, compressive modulus, and compressive strength for laminates P1–P4. Further supported by the DSC results provided in Figure S6, laminate P1 exhibits the highest mechanical properties among all laminates, making it the reference for comparison. Its thermal behavior, with a Tg nearly identical to that of fully cured epoxy resin, reflects a highly cross-linked polymer network, which directly contributes to superior interlaminar shear strength (ILSS) and compressive performance. In fact, the superior mechanical strength and toughness of GFRP are strongly correlated with a higher degree of cure in the polymer matrix and enhanced fiber-matrix interfacial adhesion.^43,44^

**Figure 14:**
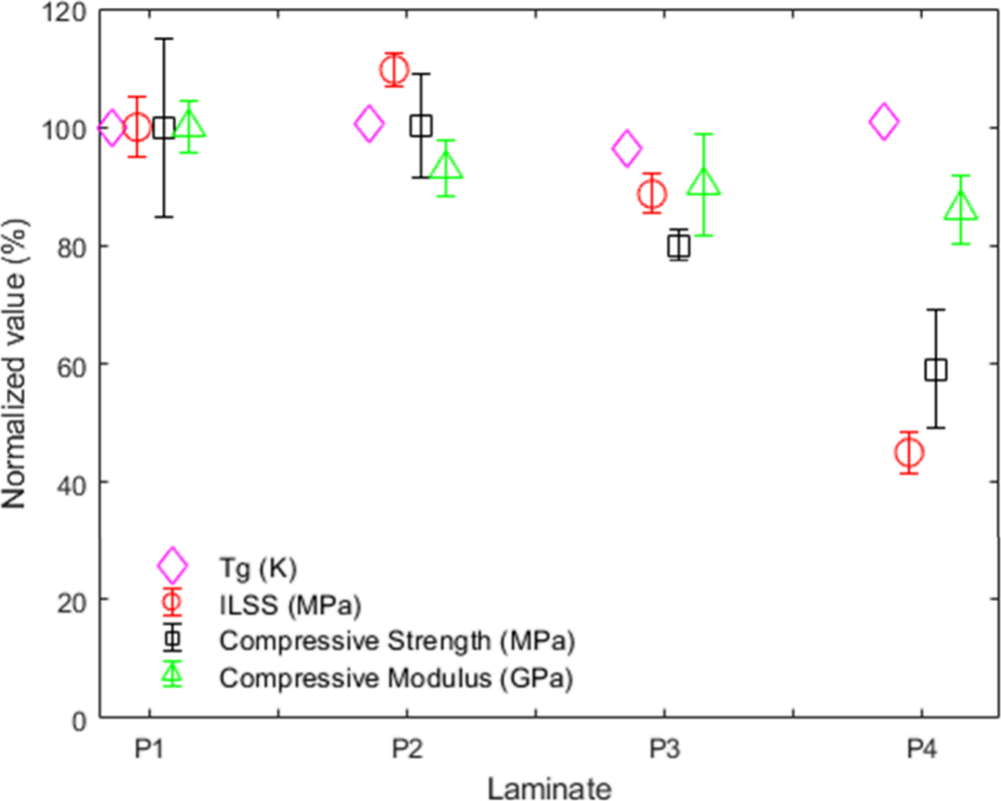
Evaluation of Tg, ILSS, and compressive properties across laminates P1–P4.

By comparison, laminate P2, which achieved full cure under the ramp cycle, showed similar properties, with a compressive modulus of 41.9 GPa, compressive strength of 443.8 MPa, and comparable ILSS of 42.9 MPa. The close agreement between P1 and P2 indicates good curing uniformity. It is noteworthy that average ILSS value obtained for samples P1 and P2 (41 MPa) belong to the standard GFRP region (40–70 MPa).^44^

The interlaminar shear strength of laminate P3 is impacted by vitrification, which can be indirectly detected through an exothermic peak in a following DSC scan (shaded green area in Figure S6c). In these conditions, the resin’s mobility is restricted due to increased crosslinking, leading to potential brittleness. This can result in a slight decrease in ILSS, as the matrix becomes less capable of dissipating energy through plastic deformation (34.7 MPa). Regarding the compressive modulus (40.6 GPa) is 9.8% lower than the fully cured P1 but 4.4% higher than the degraded sample P4 (38.8 GPa). Similarly, the compressive strength (354.8 MPa) is reduced by 19.9% compared with the fully cured laminate, yet remains 26.4% higher than P4 (261.2 MPa). This intermediate behavior results from incomplete polymerization, where early vitrification interrupts crosslinking, resulting in a semicured network with reduced properties.

Finally, at excessively high temperatures, thermal degradation becomes significant, and prolonged exposure can markedly reduce the resin’s mechanical performance. Specimen P4, subjected to these conditions, confirmed this prediction, showing a maximum shear strength reduction of 54.9% compared to the ILSS of sample P1. For this laminate (P4), no decrease in Tg was detected, suggesting that different mechanisms are competing within the system. Normally, degradation would lower Tg by increasing polymer mobility. However, this tendency can be balanced out by additional cross-linking reactions that stiffen the network structure. In addition, strong fiber-matrix interactions can further limit polymer mobility, helping to stabilize Tg. The absence of a Tg decrease therefore implies that degradation induced chain scission is balanced or overwhelmed by structural reinforcements or crosslinking, preserving the composite’s thermal stability. Indeed, sample P4 showed a 13.8% reduction in modulus (38.8 GPa) and a severe 41.0% strength loss (261.2 MPa). This relevant degradation suggests compromised fiber-matrix interfacial bonding which dominantly affects strength while the relatively smaller modulus decrease implies partial matrix plasticization. The results highlight that environmental or aging degradation primarily impacts load-bearing capacity rather than stiffness, a critical consideration for structural applications.

Indeed, sample P4 showed a 13.8% reduction in modulus (38.8 GPa) and a severe 41.0% strength loss (261.2 MPa). This degradation suggests that multiple mechanisms may be involved, including possible fiber-matrix interfacial deterioration, which typically has a stronger effect on strength than stiffness. The relatively smaller decrease in modulus may indicate that the bulk matrix stiffness was less affected; however, thermooxidative effects could also contribute, potentially leading to localized surface oxidation and stiffness changes during prolonged exposure at elevated temperature.^45^ Such competing mechanisms may coexist with matrix aging and interfacial damage. Overall, the results indicate that environmental or thermal aging primarily reduces load-bearing capacity rather than elastic stiffness, which is a critical consideration for structural applications.

## 4 Conclusion

This work demonstrates that conventional isothermal TTT diagrams are inadequate for describing realistic industrial cure cycles involving a prior heating ramp: for heating rates representative of industrial autoclave processing (2 °C/min), the degree of cure accumulated during the heating ramp exceeds 10% across the entire practical curing temperature range. To address this limitation, a TTT-β framework was developed to explicitly incorporate the influence of nonisothermal preheating on curing and degradation behavior in DGEBA–amine systems.

Continuous heating curing achieved nearly identical mechanical performance to ramp–isotherm treatment in a shorter overall process time: ILSS of 42.9 MPa vs 39.1 MPa, compressive strength of 443.8 MPa vs 442.8 MPa, and compressive modulus of 41.9 GPa vs 45.0 GPa. These results demonstrate that equivalent mechanical performance can be achieved in a reduced cycle time by eliminating the isothermal hold.

Vitrification reduced ILSS by 11.3% (34.7 MPa), compressive strength by 19.9% (354.8 MPa), and compressive modulus by 9.8% (40.6 GPa). These reductions are consistent with incomplete crosslinking that preferentially impairs matrix-dominated and interface-sensitive properties.

Severe degradation produced substantially larger losses: ILSS decreased by 54.9% (17.6 MPa) and compressive strength by 41.0% (261.2 MPa), while the compressive modulus decreased by only 13.8% (38.8 GPa) despite a Tg of 134 °C, comparable to that of the fully cured reference. This decoupling between Tg and mechanical performance, confirms that Tg alone is insufficient as a quality indicator for processed composites, and underscores the necessity of integrating degradation criteria into the processing chart.

By integrating the effects of both heating ramp and isothermal exposure, the proposed methodology provides a more realistic and industrially relevant framework for cure cycle optimization and for achieving target material properties in composite manufacturing applications.

## Supplementary Material



## Notes

Interlaminar shear strength (ILSS) experimental setup, compression test specimens with strain gauges, compression test fixture, heats of reaction at different heating rates, DSC curves of samples (curing process and postcuring Tg analysis), load vs displacement curves of epoxy/glass fiber composites under different curing cycles, DSC scans of laminates P1–P4 after curing, specimen dimensions and ILSS values of glass fiber composites, compressive stress–strain curves under different curing cycles, specimen dimensions for compression tests (PDF)
