# Supplementary material for: Incorporating the Heating Stage into Processability Maps for Epoxy Curing Optimization
Source: ACS Omega. 2026 Jul 7;11(28):42131–42. doi: 10.1021/acsomega.6c02541 (PMC13392904; doi:10.1021/acsomega.6c02541)
Supplement: Supplementary file 1 [file ao6c02541_si_001.pdf]

Supplementary materials

Incorporating the Heating Stage into Processability Maps for Epoxy Curing Optimization

Sihem Zaidi[1,2*], Daniel Sánchez-Rodriguez[1,2*], Ahmed Mohamed Saleh Abd Elfatah[1,3], Jordi Farjas[1,2], Paul Spencer[4], Josep Costa[1]

[1]AMADE - Analysis and Advanced Materials for Structural Design, Polytechnic School, University of Girona, C/ Universitat de Girona 4, E-17003 Girona, Spain.

[2]GRMT - Materials Research Group and Thermodynamics, Polytechnic School, University of Girona, C/ Universitat de Girona 4, E-17003 Girona, Spain.

[3]Materials Engineering Department, Faculty of Engineering, Zagazig University, Zagazig 44519, Egypt

[4]Gurit - Gurit (UK) Ltd, St Cross Business Park, Newport, Isle of Wight, PO30 5WU, United Kingdom

**Corresponding author: sihem.zaidi@udg.edu, Tel. +34 972419757*

daniel.sanchez@udg.edu, Tel. +34 972419757

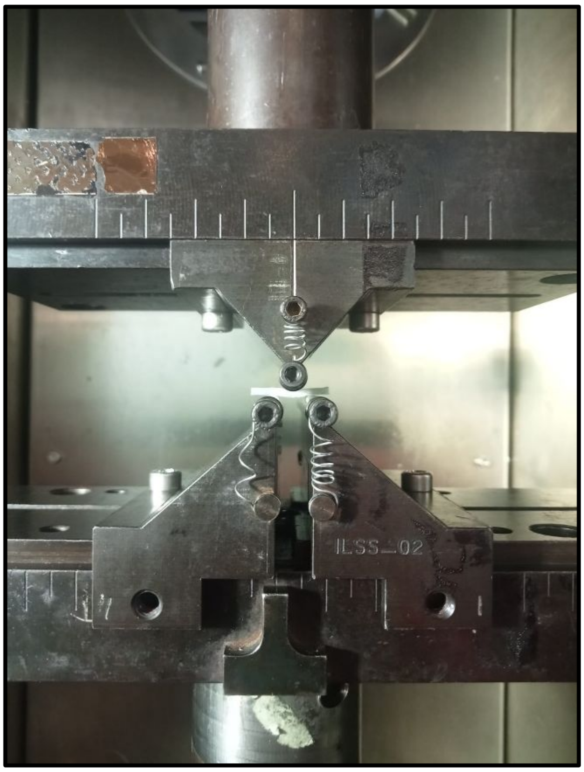

Fig. S1. Experimental setup of Interlaminar shear strength (ILSS)

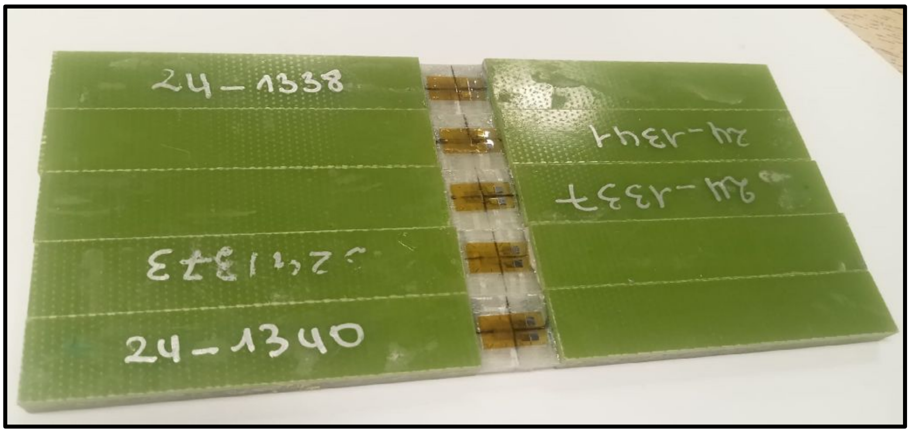

Fig. S2. Compression test specimens with strain gages

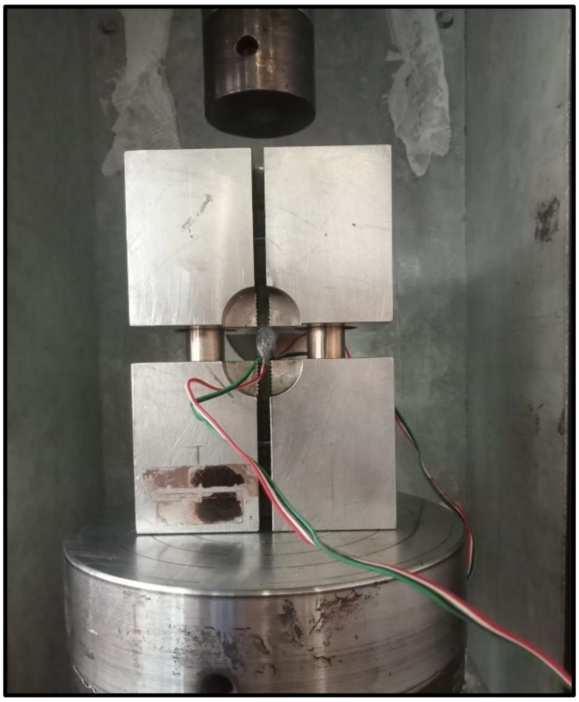

Fig. S3. Test fixture for compression

Table S1. The heats of reaction at different heating rates.

Heating ramp [ºC/min]	Enthalpy [J/g]
20	430.9
10	420.7
5	448.4
2.5	458.9
1.25	433

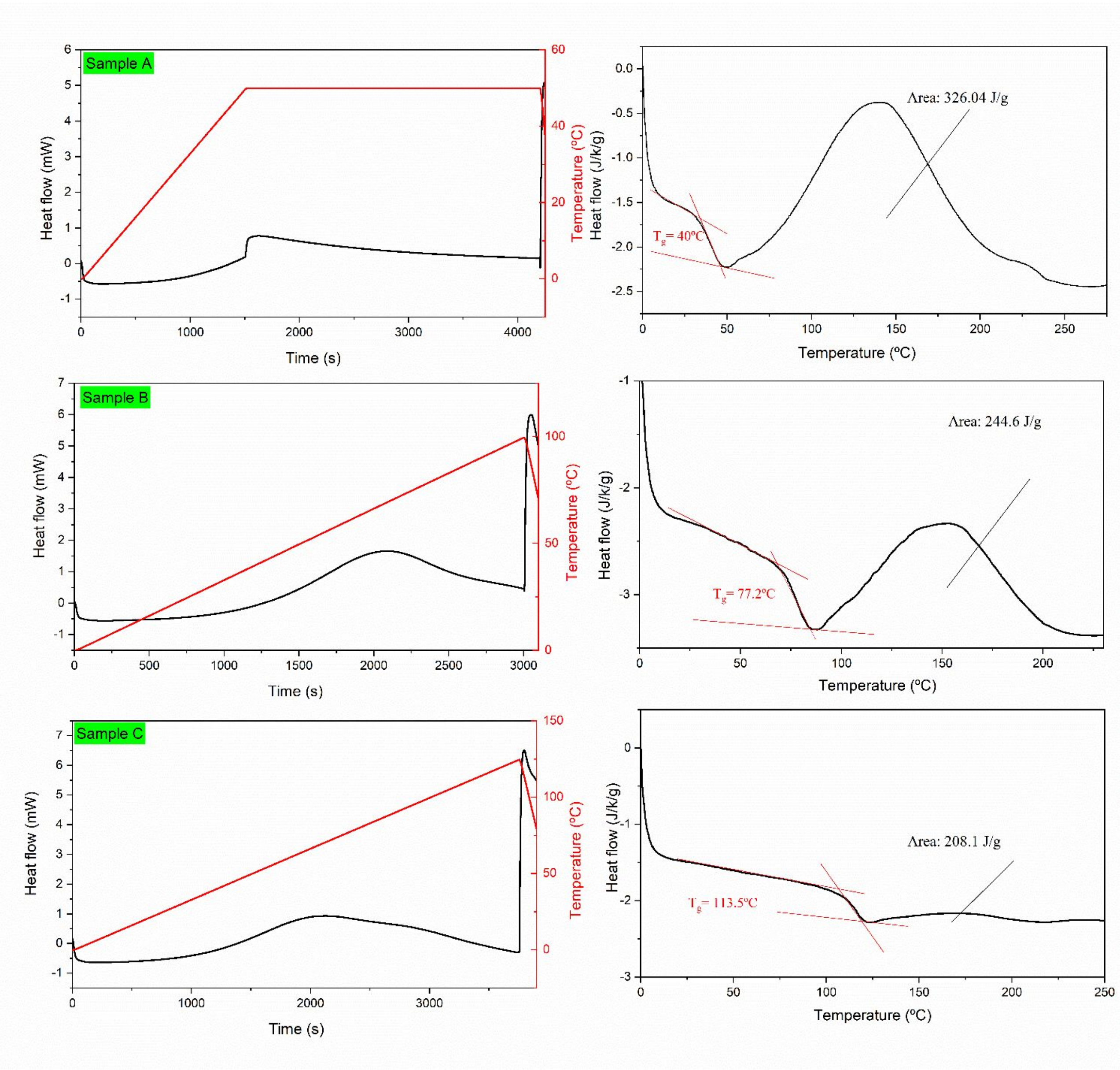

Fig. S4. DSC curves of the samples listed in Table 2: The figures on the left side correspond to the curing process, while the curves on the right side were obtained afterward to determine the glass transition temperature (Tg) and the enthalpy required to complete the curing process.

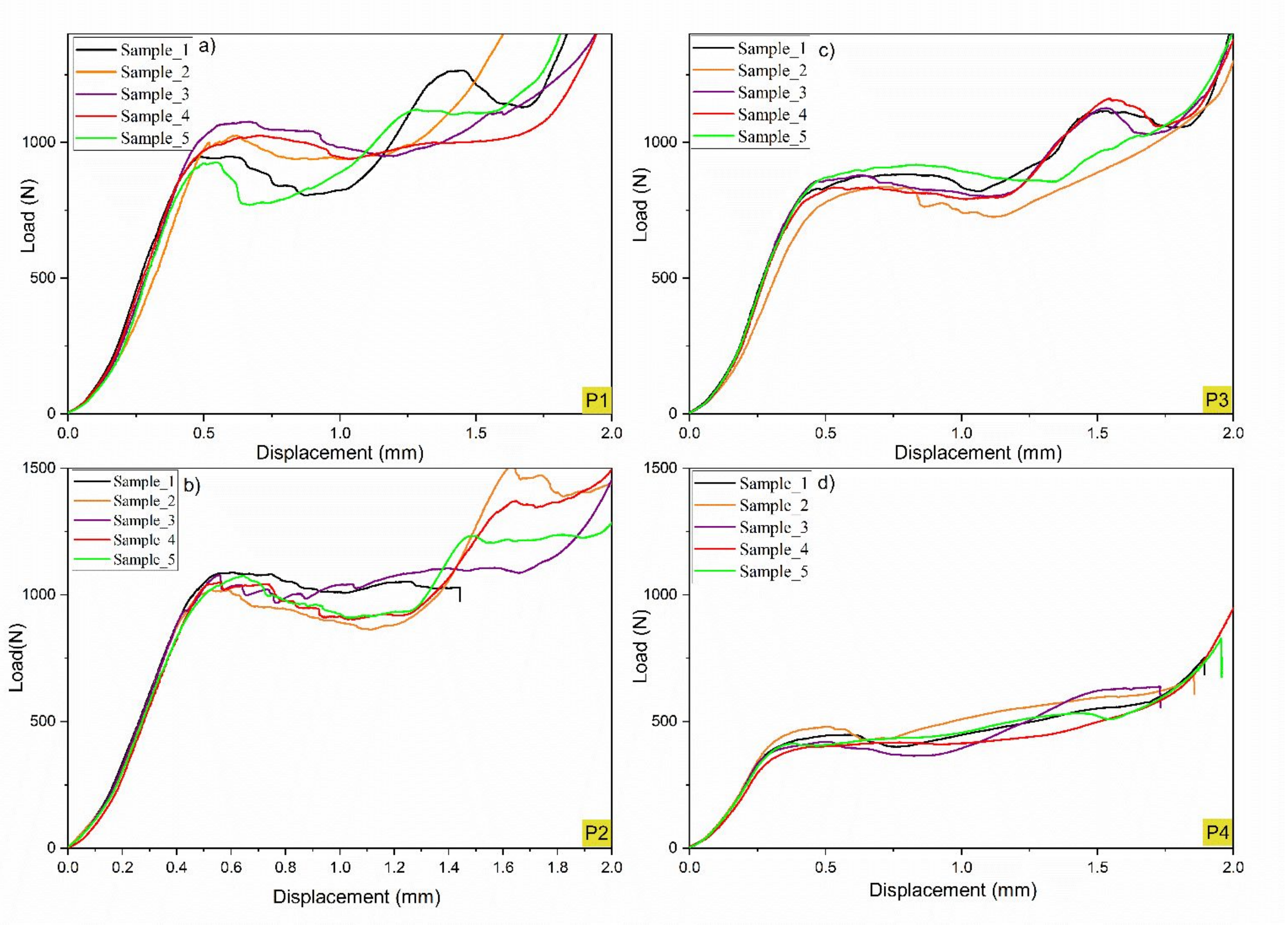

Fig. S5. Load vs displacement curves of epoxy/glass fiber composites of samples treated at different curing cycles.

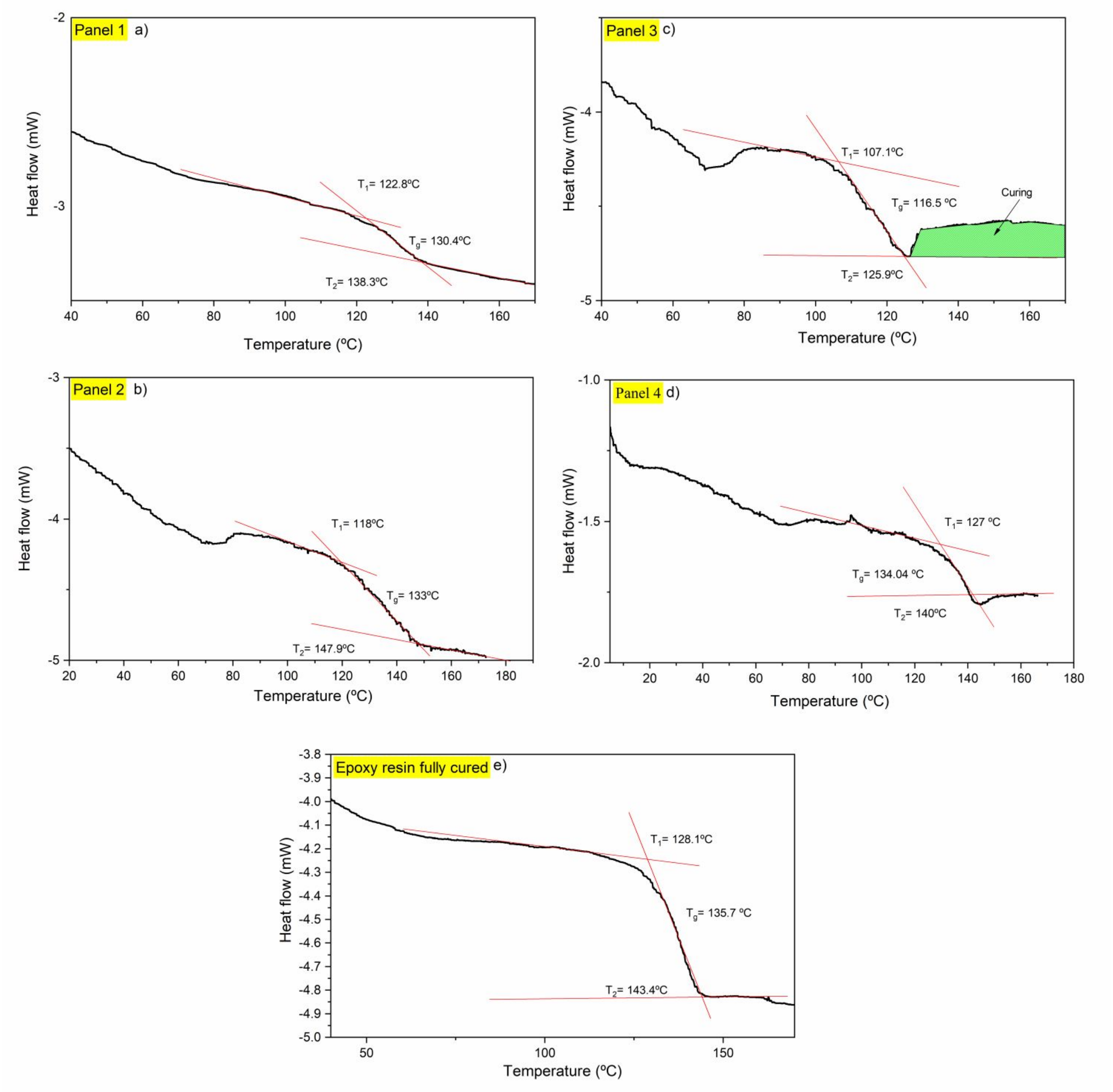

Fig. S6. DSC scans of the laminates P1, P2, P3 and P4 after curing.

Table S2. Dimensions of specimens and the values of ILSS of the glass fiber.

Panel	Sample	Thickness [mm]	Width[mm]	Length [mm]	Max applied load [N]	ILSS [MPa]
**P1**	Sample_1	1.88	10.03	20.10	944.04	37.54
	Sample_2	1.89	10.05	20.11	1001.3	39.53
	Sample_3	1.89	10.05	20.08	1064.39	42.02
	Sample_4	1.86	10.06	20.11	991.37	39.73
	Sample_5	1.87	10.06	20.14	928.3	37
**Average**						39.1
**P2**	Sample_1	1.85	10.01	20.15	1086.46	44
	Sample_2	1.83	10.04	20.17	1015.32	41.44
	Sample_3	1.83	10.05	20.12	1078.97	44
	Sample_4	1.84	10.04	20.16	1048	42.54
	Sample_5	1.82	10.04	20.13	1039.15	42.65
**Average**						42.9
**P3**	Sample_1	1.83	10.01	20.15	871.90	35.79
	Sample_2	1.85	10.05	20.34	821.51	33.13
	Sample_3	1.87	10.06	20.19	877.83	34.99
	Sample_4	1.84	10.07	20.14	830.4	33.61
	Sample_5	1.86	10.07	20.14	298.58	36.01
**Average**						34.7
**P4**	Sample_1	1.83	10.09	20.16	446.94	18.5
	Sample_2	1.85	10.07	20.19	477.68	19.23
	Sample_3	1.82	10.07	20.12	421.31	17.24
	Sample_4	1.86	10.06	20.13	403.38	16.16
	Sample_5	1.80	10.08	20.19	408.50	16.88
**Average**						17.6

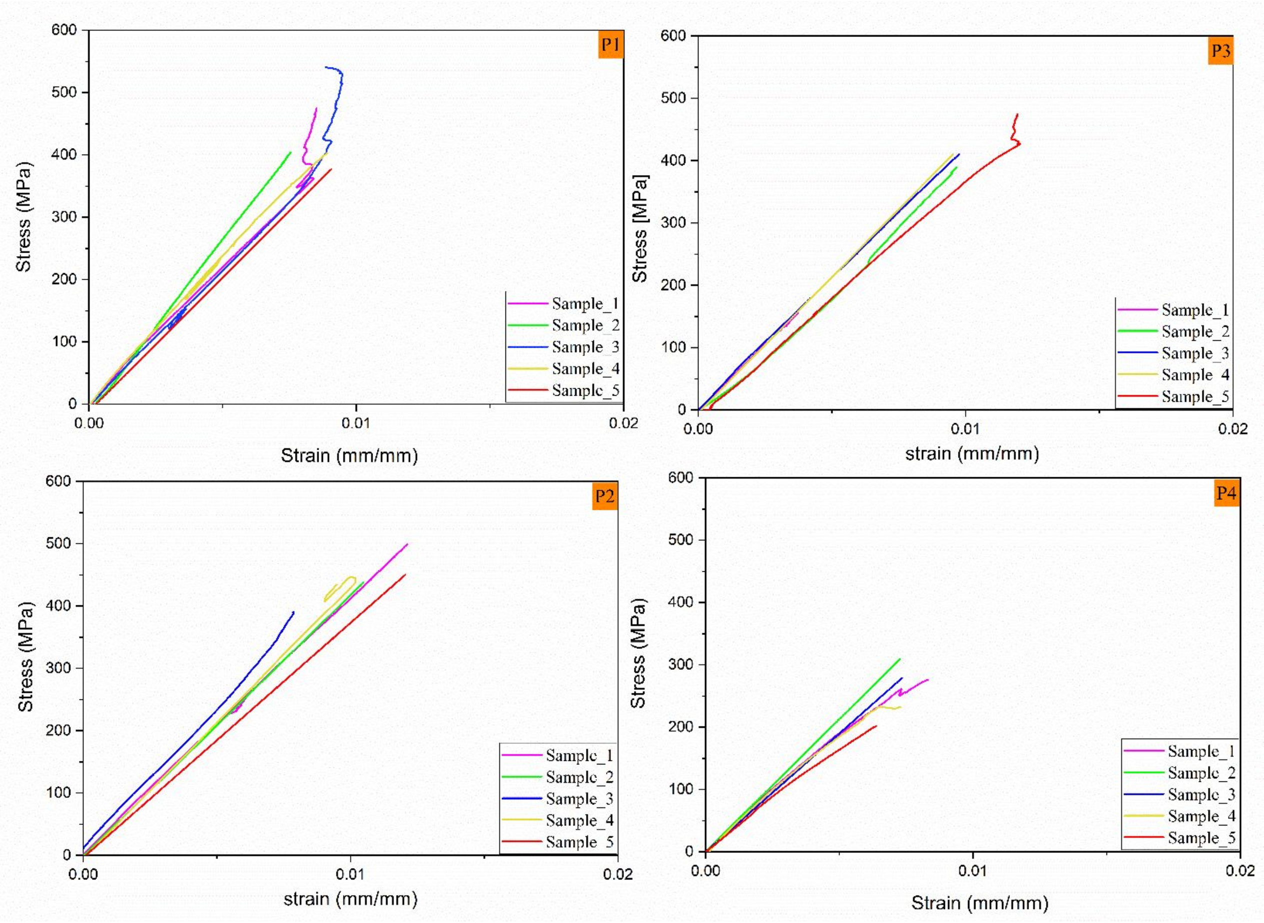

Fig. S7. Compressive stress vs strain curves of epoxy/glass fiber composites treated at different curing cycles

Table S3. Specimens dimensions used for compression tests

Panel	Sample	Thickness [mm]	Width[mm]	Length [mm]	Compressive modulus [GPa]	Compressive strength [MPa]
	Sample_1	2.02	13.12	140.08	44.41	474
	Sample_2	2.02	13.10	140.10	47.28	426
**P1**	Sample_3	2.01	13.14	140.13	43.17	541
	Sample_4	2.02	13.12	140.07	47.01	404
	Sample_5	1.99	13.13	140.19	43.32	369
**Average**					45.03	442.8
	Sample_1	1.99	13.13	140.10	42.48	499
	Sample_2	2.00	13.15	140.12	42.20	434
**P2**	Sample_3	2.00	13.12	140.13	43	491
	Sample_4	2.01	13.13	140.15	43.42	446
	Sample_5	1.99	13.13	140.14	38.30	449
**Average**					41.88	443.8
	Sample_1	2.00	13.14	140.13	41.50	156
	Sample_2	2.00	13.14	140.15	35.31	389
**P3**	Sample_3	2.00	13.14	140.08	42.61	411
	Sample_4	2.01	13.12	140.12	45.37	408
	Sample_5	1.99	13.15	140.10	38.20	410
**Average**					40.59	354.8
	Sample_1	2.00	13.14	140.20	39.25	276
	Sample_2	2.02	13.10	140.20	42.46	316
**P4**	Sample_3	2.00	13.14	140.25	37.97	279
	Sample_4	1.95	13.24	140.35	38.76	233
	Sample_5	2	13.2	140.23	35.35	202
**Average**					38.75	261.2
